# Tricuspid edge-to-edge repair for tricuspid valve prolapse and flail leaflet: feasibility in comparison to patients with secondary tricuspid regurgitation

**DOI:** 10.1093/ehjci/jead264

**Published:** 2023-10-20

**Authors:** Varius Dannenberg, Philipp E Bartko, Martin Andreas, Anna Bartunek, Arseniy Goncharov, Muhammed Gerçek, Kai Friedrichs, Christian Hengstenberg, Volker Rudolph, Maria Ivannikova

**Affiliations:** Department for Internal Medicine II, Cardiology, Medical University of Vienna, Vienna, Austria; Department for Internal Medicine II, Cardiology, Medical University of Vienna, Vienna, Austria; Department of Cardiac Surgery, Medical University of Vienna, Vienna, Austria; Department of Cardiovascular, Cardiac, Thoracic and Vascular Anesthesia and Intensive Care, Medical University of Vienna, Vienna, Austria; Clinic for General and Interventional Cardiology/Angiology, Herz- und Diabeteszentrum NRW, Ruhr-Universität Bochum, Bad Oeynhausen, Germany; Clinic for General and Interventional Cardiology/Angiology, Herz- und Diabeteszentrum NRW, Ruhr-Universität Bochum, Bad Oeynhausen, Germany; Clinic for General and Interventional Cardiology/Angiology, Herz- und Diabeteszentrum NRW, Ruhr-Universität Bochum, Bad Oeynhausen, Germany; Department for Internal Medicine II, Cardiology, Medical University of Vienna, Vienna, Austria; Clinic for General and Interventional Cardiology/Angiology, Herz- und Diabeteszentrum NRW, Ruhr-Universität Bochum, Bad Oeynhausen, Germany; Clinic for General and Interventional Cardiology/Angiology, Herz- und Diabeteszentrum NRW, Ruhr-Universität Bochum, Bad Oeynhausen, Germany

**Keywords:** tricuspid regurgitation, primary leaflet defects, edge-to-edge repair

## Abstract

**Aims:**

Transcatheter tricuspid edge-to-edge repair (T-TEER) has gained widespread use for the treatment of tricuspid regurgitation (TR) in symptomatic patients with high operative risk. Although secondary TR is the most common pathology, some patients exhibit primary or predominantly primary TR. Characterization of patients with these pathologies in the T-TEER context has not been systematically performed.

**Methods and results:**

Patients assigned to T-TEER by the interdisciplinary heart team were consecutively recruited in two European centres over 4 years. Echocardiographic images were evaluated to distinguish between primary and secondary causes of TR. Both groups were compared concerning procedural results. A total of 339 patients were recruited, 13% with primary TR and 87% with secondary TR. Patients with primary TR had a smaller right ventricle (basal diameter 45 vs. 49 mm, *P* = 0.004), a better right ventricular function (fractional area change 45 vs. 41%, *P* = 0.001), a smaller right (28 vs. 34 cm^2^, *P* = 0.021) and left (52 vs. 67 mL/m^2^, *P* = 0.038) atrium, and a better left ventricular ejection fraction (60 vs. 52%, *P* = 0.005). The severity of TR was similar in primary and secondary TR at baseline (TR vena contracta width pre-interventional 13 ± 4 vs. 14 ± 5 mm, *P* = 0.19), and T-TEER significantly reduced TR in both groups (TR vena contracta width post-interventional 4 ± 3 vs. 5 ± 5 mm, *P* = 0.10). These findings remained stable after propensity score matching. Complications were similar between both groups.

**Conclusion:**

T-TEER confers equally safe and effective reduction of TR in patients with primary and secondary TR.

## Graphical Abstract

**Graphical Abstract:**
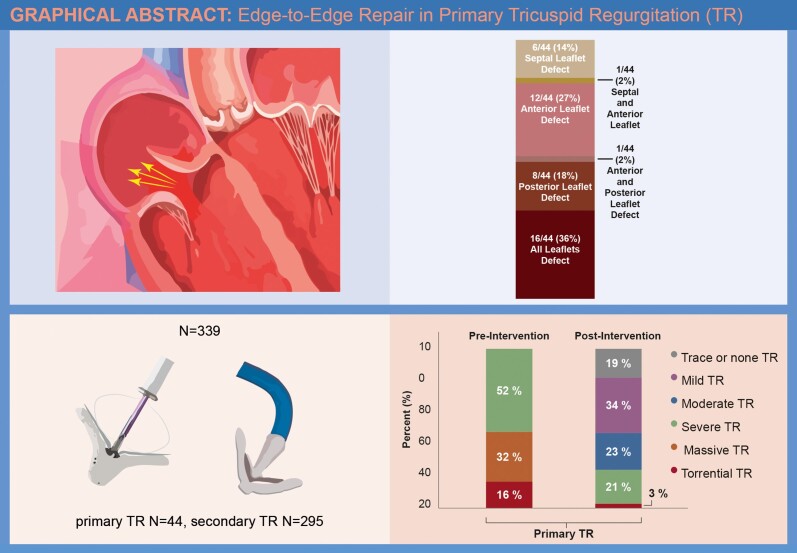
Edge-to-edge repair in patients with primary tricuspid regurgitation. TR, tricuspid regurgitation.

## Introduction

Tricuspid regurgitation (TR) is a common disease with high morbidity and mortality.^1,2^ In an overall cohort, significant TR was detected echocardiographically in 6% of all patients, of which 92.6% were secondary TR and 7.4% were primary TR.^3^ Secondary TR can be differentiated according to its underlying condition, resulting from left heart disease, right ventricular (RV) disease, pulmonary hypertension, or atrial dilatation.^4,5^ There are many reasons for primary TR. Common causes are endocarditis, cardiac implantable electronic device (CIED) leads, or rheumatic heart disease.^4,6,7^ Similar to mitral valve prolapse, the tricuspid valve (TV) may also prolapse, resulting in significant TR.^8^ Current guidelines implemented recommendations for concomitant TV surgery in patients undergoing left heart valve surgery. For isolated TR, recommendations for surgery are generally stronger for primary TR than for secondary TR.^9,10^ Interventional procedures for transcatheter tricuspid edge-to-edge repair (T-TEER) are increasingly performed and could show promising results in recent studies.^11–13^ European guidelines implemented a IIb indication for transcatheter repair for patients with symptomatic severe secondary TR for inoperable patients in experienced heart valve centres. Current guidelines do not recommend interventional therapy in patients with primary TR.^9,10^ Nevertheless, in clinical routine approved by the multidisciplinary heart team, T-TEER is often performed in inoperable patients with primary TR. These procedures have shown promising results in individual cases but have yet to be described in larger cohorts.^14^ This study focuses on T-TEER in patients with primary TR induced by leaflet defects. The main aims are to elaborate (i) the number of patients with primary leaflet defects in an all-comer cohort of T-TEER patients, (ii) their clinical characteristics compared with patients with secondary TR, and (iii) the feasibility of T-TEER and the post-procedural reduction of TR compared with patients with secondary TR.

## Methods

### Study population

Patients assigned to T-TEER were recruited at the Medical University of Vienna (Austria) and the Clinic for General and Interventional Cardiology/Angiology at the Heart & Diabetes Center NRW in Bad Oeynhausen (Germany) between September 2018 and December 2022 in a *post hoc* analysis of a prospective cohort. The multidisciplinary heart team approved each procedure according to current guidelines and careful risk stratification. Pre-procedural and peri-procedural echocardiographic images were evaluated, and patients were divided into those with secondary TR and those with primary TR. Patients with mixed aetiology were assigned to the respective group according to the predominant TR mechanism. Both groups were analysed, and the results were compared. The Ethics Committee of the Medical University of Vienna (1386/2019) and the Ruhr University Bochum (AZ 2023-1018) approved the study protocol. All patients consented to participate.

### Echocardiographic assessment

**Figure 1:**
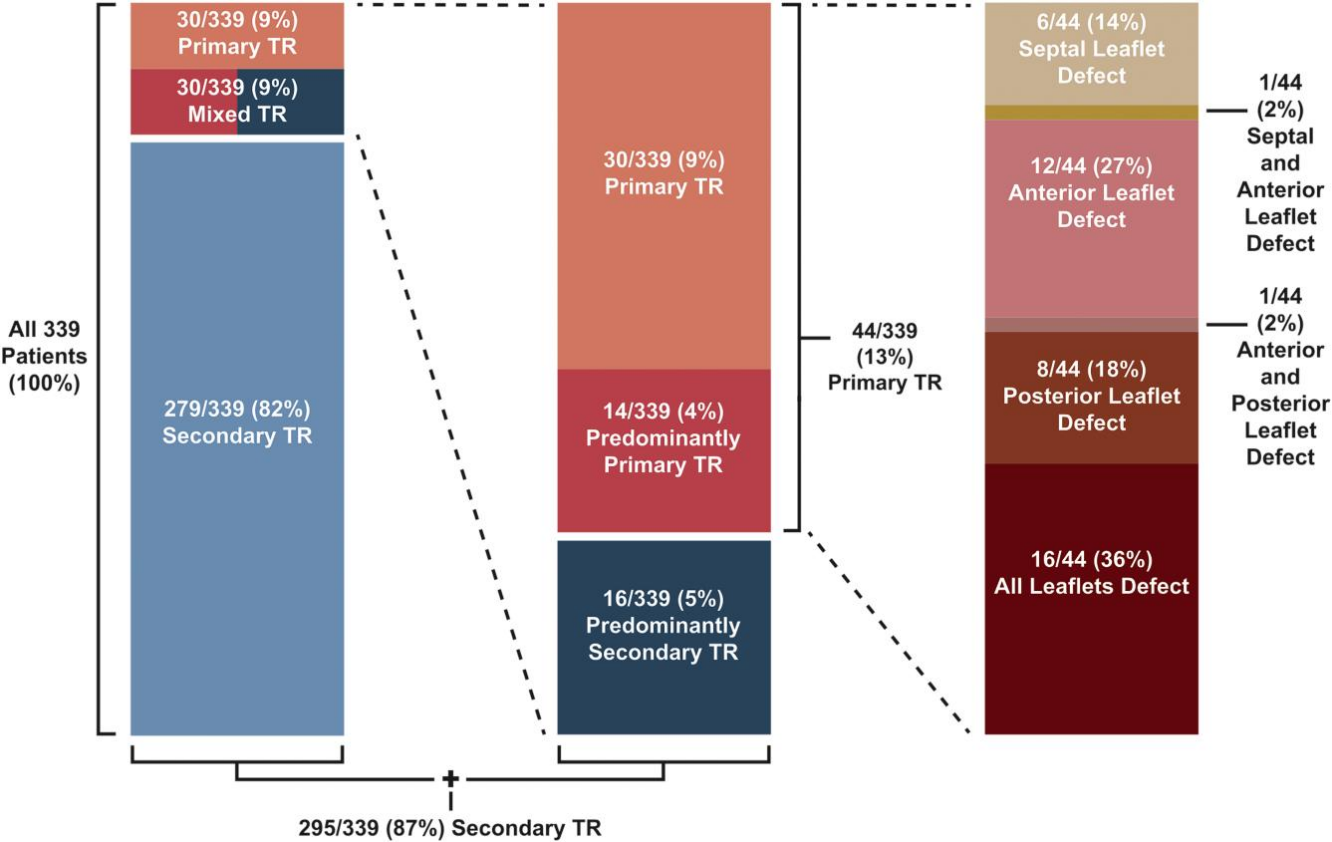
Distribution of primary and secondary tricuspid regurgitation in tricuspid edge-to-edge repair patients. TR, tricuspid regurgitation.

A comprehensive transthoracic (TTE) and transoesophageal echocardiographic (TEE) assessment was performed according to the American Society of Echocardiography and the European Society of Cardiovascular Imaging recommendations pre-, peri-, and post-procedurally.^15,16^ Physicians and sonographers examined all patients using commercially available equipment (Vivid 7, E9, E95, GE Healthcare; and EPIQ 7, Philips Medical Systems). Board-certified physicians interpreted all examinations. The comprehensive echocardiographic assessment is described elsewhere.^13^ Interventional echocardiographic specialists (M.I. and V.D.) reviewed the pre-interventional and peri-interventional echocardiograms and screened for TV prolapse (TVP) and flail TV leaflets (TVFs). Traumatic TVF due to chest trauma was excluded from the health records review. Lorinsky *et al*.^8^ recently described a normal atrial displacement of the TV of 4 mm in the RV inflow view and 2 mm in the parasternal short axis and the four-chamber view. Therefore, patients with TVP were considered primary TR patients if they exceeded these thresholds and no typical signs of secondary TR were present or if they presented with TVF. If signs of secondary TR were present in addition to TVP above the thresholds, we counted these patients as mixed aetiology TR patients. Mixed TR patients were differentiated into predominantly primary and predominantly secondary TR patients. Patients with predominantly primary and primary TR built the final group of primary TR patients, and patients with predominantly secondary and secondary TR created the final group of secondary TR patients (*Figure 1*). In patients with CIED, an interaction with the lead is carefully evaluated as part of the screening process for T-TEER. These patients are usually assigned to lead revision by the heart team. If T-TEER was performed, CIED was usually not the leading cause of TR. Therefore, these patients were assigned to the group according to the leading cause of TR. This was mostly secondary. Furthermore, we added a subgroup analysis by right heart chamber size, RV function, and pulmonary hypertension. RV and right atrial size were compared according to recently described patterns.^17^ RV dysfunction was defined as tricuspid annulus plane systolic excursion < 17 mm and suggested pulmonary hypertension by TR peak velocity > 2.8 m/s according to guideline recommendation.^16,18^ All values were compared between secondary and primary TR patients.

Post-processing was performed using GE EchoPac version 203 (GE Vingmed, Horten, Norway) and IntelliSpace Cardiovascular version 3.2 (Philips Medical Systems).

### Procedural characteristics

Transcatheter tricuspid valve repair was performed using the Tri-/MitraClip (Abbott Laboratories, North Chicago, IL, USA, size XT and XTW) or PASCAL system (Edwards Lifesciences, Irvine, CA, USA, size P10 and Ace). Procedural details are described elsewhere.^13^ Interventional physicians chose the procedural strategies for all patients according to their expertise and the individual patient’s anatomy. This study did not influence the procedural strategy or peri-procedural patient care. The following complications and post-procedure events were assessed: single leaflet device attachments, device embolization or thrombosis, major bleeding, conversion to surgery, and CIED implantation. Major bleeding was defined according to the Bleeding Academic Research Consortium type 3a or higher.^19^

### Statistical analysis

We demonstrated continuous variables as mean (standard deviation) and compared them with a two-sided Student’s *t*-test or Mann–Whitney *U* test. Categorical variables were described as frequencies and compared with Fisher’s exact test. A two-sided *P*-value of <0.05 was considered statistically significant. Propensity score matching was performed according to McMurry *et al*.^20^ Propensity scores were calculated using a multivariable logistic regression model. The matching procedure involved a 1:1 ratio, using nearest neighbour matching and a calliper width fixed at 0.05 standard deviations of the propensity score. The propensity score model was adjusted, accounting for differences in gender, age, body surface area, and atrial fibrillation. All analyses were performed using SPSS 28 (IBM SPSS, USA).

## Results

### Baseline characteristics, complications, and subgroup analysis

We recruited a total of 339 patients, 165 in Bad Oeynhausen and 174 in Vienna. The described screening process classified 44 patients (13%) as primary TR patients and 295 (87%) as secondary TR patients (*Figure 1*). In patients with primary TR, 66% were female. In patients with secondary TR, 52% were female. Symptoms between both groups were similar (New York Heart Association functional class ≥III, 90 vs. 92%, *P* ≥ 0.99, leg oedema 55 vs. 66%, *P* = 0.18), and N-terminal pro-brain natriuretic peptide (NT-pro-BNP) was higher in patients with secondary TR (2793 ± 2436 vs. 5333 ± 6550 pg/mL, *P* < 0.001) (*Table 1*). Patients with primary TR had a smaller RV basal diameter (45 ± 9 vs. 49 ± 8 mm, *P* = 0.004) and annulus (41 ± 7 vs. 43 ± 7 mm, *P* = 0.042), a better RV function (tissue Doppler imaging 11 ± 3 vs. 10 ± 3 cm/s, *P* = 0.023, tricuspid annulus plane systolic excursion 20 ± 5 vs. 17 ± 5 mm, *P* = 0.003, and fractional area change 45 ± 8 vs. 41 ± 9%, *P* = 0.001), a smaller right (30 ± 10 vs. 36 ± 12cm^2^, *P* = 0.001) and left (52 ± 15 vs. 67 ± 28 mL/m^2^, *P* = 0.038) atrium, and a better left ventricular ejection fraction (60 ± 8 vs. 52 ± 12%, *P* < 0.001) (*Table 2*). Detailed anatomical parameters for primary TR patients are in *Table 3*. Complications and post-procedure events are presented in *Table 4*. After propensity score matching, significant differences in baseline characteristics between both groups were observed in the presence of leg oedema (55 vs. 88%, *P* = 0.020), CIED (9 vs. 19%, *P* = 0.038), and the level of NT-pro-BNP (2793 ± 2436 vs. 4613 ± 4668 pg/mL, *P* = 0.046) (see Supplementary data online, *Table S1*). Echocardiographically, RV function (tissue Doppler imaging 11 ± 3 vs. 10 ± 3 cm/s, *P* = 0.040, tricuspid annulus plane systolic excursion 20 ± 5 vs. 16 ± 5 mm, *P* = 0.001, and fractional area change 45 ± 8 vs. 41 ± 10%, *P* = 0.021) and left ventricular ejection fraction (60 ± 8 vs. 51 ± 13%, *P* < 0.001) remained significantly better and the left atrium remained smaller (52 ± 15 vs. 67 ± 26 mL/m^2^, *P* = 0.039) in patients with primary TR after propensity score matching (see Supplementary data online, *Table S2*). We presented the baseline medication of primary and secondary TR patients in Supplementary data online, *Table S3*. The subgroup analysis of RV function, RV and right atrial size, and pulmonary hypertension is presented in Supplementary data online, *Table S4*. We could observe a significant difference between RV sizes and RV function. Right heart patterns differed between the groups if both chambers were enlarged or both chambers were of normal size.

**Table 1:** Baseline characteristics for primary and secondary TR patients

Parameter	Primary TR	Secondary TR	*P*-value
Age, years, mean ± SD	79 ± 8	78 ± 8	0.48
Female, *n* (%)	29 (66)	152 (52)	0.08
BSA, mean ± SD	1.8 ± 0.23	1.9 ± 0.21	**0**.**003**
BMI, mean ± SD	24 ± 4	26 ± 5	**0**.**017**
Dyspnoea, NYHA ≥III, *n* (%)	36 (90)	249 (92)	0.66
Leg oedema, *n* (%)	24 (55)	188 (66)	0.18
CAD, *n* (%)	16 (36)	143 (49)	0.15
Previous MCI, *n* (%)	4 (9)	31 (11)	>0.99
Previous PCI, *n* (%)	7 (18)	90 (32)	0.10
Previous CABG, *n* (%)	4 (9)	51 (17)	0.20
Previous valve surgery, *n* (%)	6 (14)	43 (15)	>0.99
Previous valve intervention, *n* (%)	2 (5)	32 (11)	0.28
Atrial fibrillation, *n* (%)	37 (84)	266 (90)	0.29
CIED, *n* (%)	9 (21)	105 (36)	0.06
Stroke, *n* (%)	5 (11)	39 (13)	0.82
COPD, *n* (%)	5 (11)	59 (20)	0.22
Renal failure, *n* (%)	26 (59)	187 (63)	0.62
Dialysis, *n* (%)	0 (0)	8 (3)	0.40
COD, *n* (%)	0 (0)	27 (9)	**0**.**034**
PAD, *n* (%)	3 (7)	33 (11)	0.45
Hypertension, *n* (%)	37 (84)	238 (81)	0.69
Diabetes, *n* (%)	6 (14)	73 (25)	0.13
Dyslipidaemia, *n* (%)	20 (46)	150 (51)	0.52
NT-pro-BNP, pg/mL, mean ± SD	2793 ± 2436	5333 ± 6550	**<0**.**001**
EuroSCORE II, mean ± SD	6 ± 5	8 ± 7	0.22
TRI-SCORE, mean ± SD	7 ± 6	9 ± 10	**0**.**015**

Bold values are significant.

BMI, body mass index; BSA, body surface area; CABG, coronary artery bypass graft; CAD, coronary artery disease; CIED, cardiac implantable electronic device; COD, cerebral artery occlusive disease; COPD, chronic obstructive pulmonary disease; MCI, myocardial infarction; NT-pro-BNP, N-terminal pro-brain natriuretic peptide; NYHA, New York Heart Association; PAD, peripheral artery disease; PCI, percutaneous coronary intervention; SD, standard deviation; TR, tricuspid regurgitation.

**Table 2:** Echocardiographic parameters for primary and secondary TR patients

Parameter	Primary TR	Secondary TR	*P*-value
RV basal diameter, mm, mean ± SD	45 ± 9	49 ± 8	**0**.**004**
TV annulus diameter, mm, mean ± SD	41 ± 7	43 ± 7	**0**.**042**
TAPSE, mm, mean ± SD	20 ± 5	17 ± 5	**0**.**003**
TDI, cm/s, mean ± SD	11 ± 3	10 ± 3	**0**.**023**
FAC, %, mean ± SD	45 ± 8	41 ± 9	**0**.**001**
RA area, mean ± SD	30 ± 10	36 ± 12	**0**.**001**
Estimated sPAP, mmHg, mean ± SD	46 ± 13	44 ± 14	0.40
LVEDV, ml, mean ± SD	84 ± 37	98 ± 40	**0**.**037**
LV EF, %, mean ± SD	60 ± 8	52 ± 12	**<0**.**001**
LA volume index, mL/m^2^, mean ± SD	52 ± 15	67 ± 28	**0**.**038**
TR grade pre-interventional, median (IQR)	3 [1]	4 [1]	0.12
TR grade, post-interventional, median (IQR)	1 [1]	1 [1]	0.47
Residual TR grade ≤2, *n* (%)	33 (77)	228 (78)	0.85
Delta TR grade, median (IQR)	2 [2]	2 [2]	0.94
TR VC, pre-interventional, mm, mean ± SD	13 ± 4	14 ± 5	0.19
TR VC, post-interventional, mm, mean ± SD	4 ± 3	5 ± 5	0.10
Delta VC, mean ± SD	9 ± 5	9 ± 6	0.96
TR Vmax, m/s, mean ± SD	3.0 ± 0.5	2.9 ± 0.7	0.34
TR EROA, mm^2^, mean ± SD	62 ± 33	68 ± 40	0.32
TR RegVol, mL, mean ± SD	57 ± 26	57 ± 22	0.99

Bold values are significant.

EROA, effective regurgitation orifice area; FAC, fractional area change; LA, left atrium; LV EF, left ventricular ejection fraction; LVEDV, left ventricle end-systolic volume; RA, right atrium; RegVol, regurgitation volume; RV, right ventricle; SD, standard deviation; sPAP, systolic pulmonary artery pressure; TAPSE, tricuspid annulus plane systolic excursion; TDI, tissue Doppler imaging; TR, tricuspid regurgitation; TV, tricuspid valve; VC, vena contracta width.

**Table 3:** Detailed anatomical and procedural parameters for primary TR patients

Parameter	Primary TR
Prolapse, *n* (%)	29 (66)
Prolapse gap, mean ± SD	5 ± 1.4
Flail, *n* (%)	16 (36)
Flail gap, mean ± SD	5 ± 2.7
Dysjunction, *n* (%)	9 (21)
Location defect septal leaflet, *n* (%)	6 (16)
Location defect septal and anterior leaflet, *n* (%)	1 (2)
Location defect anterior leaflet, *n* (%)	12 (27)
Location defect anterior and posterior leaflet, *n* (%)	1 (2)
Location defect posterior leaflet, *n* (%)	8 (18)
Location defect posterior and septal leaflet, *n* (%)	0 (0)
All leaflets defect, *n* (%)	16 (36)
Number devices anteroseptal, mean ± SD	0.9 ± 0.7
Number devices posteroseptal, mean ± SD	0.7 ± 0.7
Number devices anteroposterior, mean ± SD	0.1 ± 0.2
Number devices implanted per patient, mean ± SD	1.7 ± 0.8

SD, standard deviation; TR, tricuspid regurgitation.

**Table 4:** Complications and post-procedure events

Parameter	Primary TR	Secondary TR	*P*-value
SLDA, *n* (%)	1 (2)	14 (5)	0.70
Device embolization or thrombosis, *n* (%)	0	0	>0.99
Major bleeding, *n* (%)	1 (2)	5 (2)	>0.99
Conversion to surgery, *n* (%)	0	0	>0.99
CIED implantation, *n* (%)	0	1 (0)	>0.99

CIED, cardiac implantable electronic device; SLDA, single leaflet device attachment; TR, tricuspid regurgitation.

### Reduction of TR

**Figure 2:**
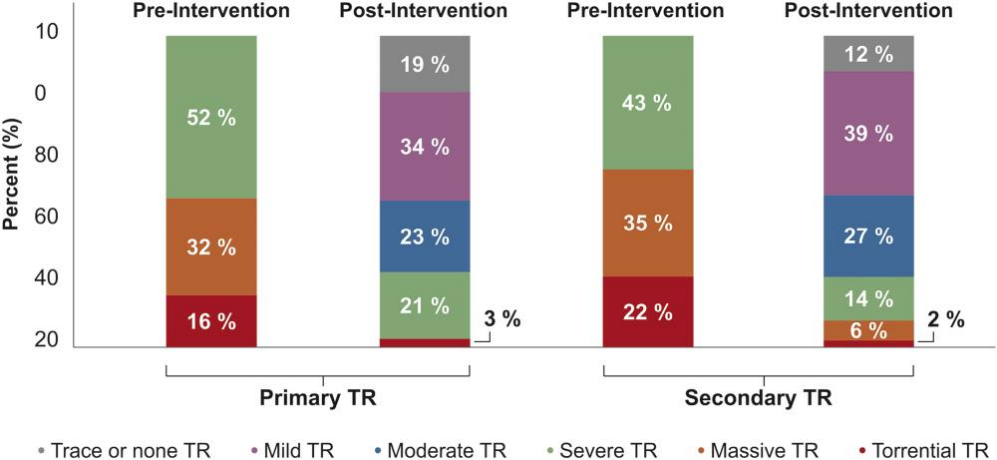
Interventional results of tricuspid edge-to-edge repair in patients with primary and secondary tricuspid regurgitation. TR, tricuspid regurgitation.

The severity of TR showed no difference between both groups at baseline regarding vena contracta width (13 ± 4 vs. 14 ± 5 mm, *P* = 0.19), effective regurgitation orifice area (EROA, 62 ± 33 vs. 68 ± 40mm^2^, *P* = 0.32), regurgitation volume (RegVol, 57 ± 26 vs. 57 ± 22 mL, *P* = 0.99), and TR grade [median 3 (1) vs. 4 (1), *P* = 0.12]. T-TEER could significantly reduce TR in both groups, regarding delta vena contracta width (9 ± 5 vs. 9 ± 6 mm, *P* = 0.96), delta TR grade [median 2 (2) vs. 2 (2), *P* = 0.94], and residual TR grade ≤ 2 (76 vs. 78%, *P* = 0.85) (*Table 2*; *Figure 2*). These findings remained stable after propensity score matching. In the group of primary TR patients, 36% had a TVF, and 66% had a TVP. Single leaflet defects occurred most frequently within the anterior leaflet (27%), and in 36%, all leaflets were affected by a defect (*Table 3*).

## Discussion

In this international dual-centre study, we examined patients treated with T-TEER and compared the interventional results between patients with primary TR and secondary TR. We present the following major findings: (i) in 13% of patients treated with T-TEER, a primary leaflet defect is the main underlying aetiology; (ii) patients with primary TR are more frequently female (66%), have lower overall morbidity, and show less remodelling of all heart chambers than patients with secondary TR; and (iii) for the first time, we demonstrated that T-TEER is a safe and effective therapy to achieve a substantial reduction in TR in patients with primary leaflet defects.

### Definition of primary TR

In the existing literature, various causes of primary TR are reported, affecting very different groups of patients. Known reasons for primary TR are endocarditis, Ebstein’s disease, device lead induced, neuroendocrine tumours, or iatrogenic after biopsy. Further possible reasons are rheumatic valve disease or traumatic causes.^4,6,7,21,22^ Many of these patients are usually young and are mostly sooner or later treated with surgical valve repair or valve replacement and, therefore, are not usually assigned to T-TEER.^23^ Patients referred to T-TEER by the interdisciplinary heart team are generally older and have high peri-operative mortality. When these patients have primary TR, the cause is often degenerative and includes TVP and TVF. TVFs are described as idiopathic in surgical cohorts and defined as loose leaflets with their tips pointing toward the atrium.^24,25^ The definition of TVF is relatively straightforward, but the definition of TVP is more complex. TVP has been scarcely studied but has recently become the focus of scientific debate. In a large retrospective all-comers study, the echocardiograms of 118 442 individuals were analysed, and 410 individuals were found to have suspected TVP (0.3%).^8^ Lorinsky *et al*. found that normal atrial displacement of the TV can be 4 mm in the RV inflow view and 2 mm in the parasternal short axis view and the four-chamber view. Therefore, TVP can be defined as >4 mm in the RV inflow view and >2 mm in the parasternal short axis and four-chamber view. We followed this definition and measured TVP accordingly. In a recent analysis by Guta *et al*., 465 patients with mitral valve disease and 41 healthy volunteers underwent cardiac magnetic resonance to detect and define TVP. They suggest a definition for TVP of >3 mm for the septal TV leaflet and >2 mm for the anterior and posterior TV leaflet.^26^ Although different imaging modalities are difficult to compare, the echocardiographic definition of TVP by Lorinsky *et al*., which we used, appears to be more conservative. This suggests that more patients with borderline TVP might be classified as primary TR. In the literature, primary TR accounts for ∼6–10% of patients with significant TR.^1,8,27^ In our cohort, 13% of the patients were assigned to the final group of primary TR patients. However, all T-TEER patients received a precise screening TEE. This study’s central focus is the mechanism of TR and the valve’s structural behaviour. Previous extensive studies to distinguish between primary and secondary TR rely on TTE. In TVP, patients with mixed TR could also complicate differentiation. In these patients, features of secondary TR may become apparent because primary TR can lead to right heart overload and subsequent tricuspid annular dilatation. However, this process can also lead to leaflet redundancy, which reduces annular displacement and can mask initial primary defects. Because differentiation between TVP and secondary TR might be challenging, we distinguished between predominantly primary and predominantly secondary TR patients and added them to the primary and secondary TR patients (*Figure 1*).

### Origin of the primary defects

Interestingly, 36% of primary TR patients had a defect in all TV leaflets, whereas only two patients had two leaflets affected. Single leaflet defects occurred in 59% of the patients (*Table 3*; *Figure 1*). These data rely on TVP and TVF as the mechanism for primary TR. Defects of single leaflets are more likely to be caused by TVF, whereas in patients with a defect of all leaflets, TVP is more likely to be the underlying pathology.

In a recent paediatric study of patients with Marfan’s disease, TVP occurred in 68% of the patients and correlated with mitral valve prolapse.^28^ Nevertheless, an association between connective tissue disease and TVP is difficult to extrapolate to our cohort because of the significant difference in age, time of disease onset, and co-morbidities between the two cohorts.

### Characteristics of patients with primary and secondary TR

We also observed more female patients in the primary TR cohort than in the secondary TR cohort (66% vs. 52%, *Table 1*). Lorinsky *et al*.^8^ also reported 66% of female patients in the TVP cohort compared with 50% in the non-TVP cohort. In a large global burden of disease analysis, women were found to be 27% more likely to have degenerative mitral valve disease than men. Comprehensive data for degenerative TV disease are unavailable.^29^

TR reduction in primary TR patients was similar to that in secondary TR patients. A decrease to TR ≤ 2 was achieved in 76% of primary TR patients and 78% of secondary TR patients (*Table 2*; *Figure 2*). These numbers are lower than in the recently published randomized TRILUMINATE pivotal trial, in which 83% of patients had a TR ≤ 2, and also lower than in the single-arm TriCLASP trial, in which 90% had a residual TR ≤ 2, 30 days after the intervention.^11,30^ The learning curves of the centres can explain this difference; our study included all T-TEER patients from the beginning of the program. Previous T-TEER data also report more residual TR. In the single-arm TRILUMINATE trial, 57% of patients had residual TR ≤ 2 at 30 days,^31^ and in the recently published bRIGHT study, 77% of the patients had TR ≤ 2 at 30 days.^32^ This better reflects real-world data. To view it from a different perspective, in our study and the previously mentioned studies, which include real-world data, about a quarter of the patients were left with at least severe TR after the procedure. A conservative approach with short-term follow-up or a surgical or interventional TV replacement may be considered for further treatment. In the case of interventional TV replacement, the only commercially available solution after edge-to-edge repair is currently bicaval valve implantation.^33^

Due to the significant difference between the sizes of the two groups, we performed a propensity score matching analysis based on several baseline parameters that could introduce bias into the data. In a recently published study, atrial fibrillation is a driving factor that can worsen TR. Approximately one-third of the patients developed at least moderate TR after the new onset of atrial fibrillation in an observational period of over 10 years.^34^ The authors were able to show that TR further increased mortality. We included atrial fibrillation in the propensity score matching to keep atrial fibrillation as a reason for TR similar in both groups. Furthermore, we added body surface area and gender to the analysis because the primary TR group had a more significant proportion of female patients with smaller body surface areas, which may explain the differences in the size of the ventricles. In addition, age was considered a significant factor in overall morbidity and was therefore included in the analysis. After analysis, our results of smaller and better functioning ventricles in the primary TR group remained stable.

### Clinical implications

Currently, only surgical treatment recommendations exist for patients with primary TR.^9,10^ Now that the results of the TRILUMINATE pivotal trial have been published, T-TEER recommendation in secondary TR may be expanded for symptomatic patients. Therefore, TR patients with TVF or TVP might also receive more attention in routine clinical practice. Particularly, TVF patients could have a substantial benefit from T-TEER. There is an unmet treatment demand for patients with primary leaflet defects, as they are already being treated due to the high burden of the disease. Still, no data-driven treatment recommendation can currently be formulated. These patients may also have a larger window of opportunity for treatment because of less remodelled hearts.

Complications and post-procedure events were comparable with recently published trials^11^ and did not differ significantly between primary and secondary TR (*Table 4*). T-TEER appears to be as safe in primary TR as in secondary TR. However, further studies are needed to investigate the outcome to pave the way for a future guideline recommendation.

### Limitations

Our study is limited by the evaluation of TR, which might be difficult in mixed TR patients and patients after T-TEER. Moreover, the small number of patients with primary TR might influence the statistical analysis. Furthermore, no echocardiography core laboratory was involved in image evaluation. We also did not provide outcome data in this first study of T-TEER in primary TR.

## Conclusion

Primary TR patients have lower overall morbidity but a similar burden of disease. Tricuspid edge-to-edge is equally safe and effective in both primary TR and secondary TR.

## Supplementary data

Supplementary data are available at *European Heart Journal - Cardiovascular Imaging* online.

## Supplementary Material

jead264_Supplementary_Data

## Data Availability

The data underlying this article will be shared on reasonable request to the corresponding author.
