# Supplementary material for: Tricuspid edge-to-edge repair for tricuspid valve prolapse and flail leaflet: feasibility in comparison to patients with secondary tricuspid regurgitation
Source: Eur Heart J Cardiovasc Imaging. 2023 Oct 20;25(3):365–72. doi: 10.1093/ehjci/jead264 (PMC10883724; doi:10.1093/ehjci/jead264)
Supplement: jead264_Supplementary_Data [file jead264_supplementary_data.docx]

| Supplementary Table 1 - Baseline characteristics for primary and secondary TR patients after propensity score matching for gender, age, atrial fibrillation, and body surface area | | | |
| --- | --- | --- | --- |
| Matching parameter | **Primary TR** | **Secondary TR** | **P value** |
| Age, years, mean ± SD | 79 ± 8 | 80 ± 7 | 0.30 |
| Female, n (%) | 29 (66) | 23 (52) | 0.28 |
| BSA, mean ± SD | 1.8 (0.23) | 1.8 (0.23) | 0.25 |
| Atrial fibrillation, n (%) | 37 (84) | 37 (84) | >0.99 |
| Propensity score, mean ± SD | 0.84 (0.08) | 0.85 (0.08) | 0.59 |
| Other parameter | **Primary TR** | **Secondary TR** | **P value** |
| BMI, mean ± SD | 24 ± 4 | 24 ± 4 | 0.88 |
| Dyspnea, NYHA ≥III, n (%) | 36 (90) | 41 (95) | 0.42 |
| Leg edema, n (%) | 24 (55) | 32 (80) | **0.020** |
| CAD, n (%) | 16 (36) | 24 (55) | 0.13 |
| Previous MCI, n (%) | 4 (9) | 1 (2) | 0.36 |
| Previous PCI, n (%) | 7 (18) | 17 (39) | 0.05 |
| Previous CABG, n (%) | 4 (9) | 10 (23) | 0.14 |
| Previous valve surgery, n (%) | 6 (14) | 8 (18) | 0.77 |
| Previous valve intervention, n (%) | 2 (5) | 4 (9) | 0.68 |
| CIED, n (%) | 9 (21) | 19 (43) | **0.038** |
| Stroke, n (%) | 5 (11) | 6 (14) | >0.99 |
| COPD, n (%) | 5 (11) | 6 (14) | >0.99 |
| Renal failure, n (%) | 26 (59) | 25 (57) | >0.99 |
| Dialysis, n (%) | 0 (0) | 2 (5) | 0.49 |
| COD, n (%) | 0 (0) | 3 (7) | 0.24 |
| PAD, n (%) | 3 (7) | 8 (18) | 0.20 |
| Hypertension, n (%) | 37 (84) | 35 (80) | 0.78 |
| Diabetes, n (%) | 6 (14) | 7 (16) | >0.99 |
| Dyslipidemia, n (%) | 20 (46) | 22 (50) | 0.83 |
| NT-pro-BNP, pg/ml, mean ± SD | 2793 ± 2436 | 4613 ± 4668 | **0.046** |
| EuroSCORE II, mean ± SD | 6 ± 5 | 9 ± 11 | 0.187 |
| TRI-SCORE, mean ± SD | 7 ± 6 | 5 ± 4 | 0.173 |

**TR,** tricuspid regurgitation; **BSA,** body surface area; **BMI**, body mass index; **NYHA,** New York Heart Association functional class; **CAD,** coronary artery disease; **MCI,** myocardial infarction; **PCI,** percutaneous coronary intervention; **CABG,** coronary artery bypass graft; **CIED**, cardiac implantable electronic device; **COPD,** chronic obstructive pulmonary disease; **COD,** cerebral artery occlusive disease; **PAD,** peripheral artery disease; **NT-pro-BNP,** N-terminal brain natriuretic peptide; **Bold** values are significant.

| **Supplementary Table 2 - Echocardiographic parameters for primary and secondary TR patients after propensity score matching for gender, age, atrial fibrillation, and body surface area** | | | |
| --- | --- | --- | --- |
| Parameter | **Primary TR** | **Secondary TR** | **P value** |
| RV basal diameter, mm, mean ± SD | 45±9 | 49±9 | 0.05 |
| TV annulus diameter, mm, mean ± SD | 41±7 | 44±8 | 0.08 |
| TAPSE, mm, mean ± SD | 20±5 | 16±5 | **0.001** |
| TDI s’, cm/s, mean ± SD | 11±3 | 10±3 | **0.040** |
| FAC, %, mean ± SD | 45±8 | 41±10 | **0.021** |
| RA area, mean ± SD | 30±10 | 33±10 | 0.14 |
| Estimated sPAP, mmHg, mean ± SD | 46±13 | 46±17 | 0.89 |
| LVEDV, ml, mean ± SD | 84±37 | 92±42 | 0.35 |
| LV EF, %, mean ± SD | 60±8 | 51±13 | **<0.001** |
| LA Volume index, ml/m², mean ± SD | 52±15 | 67±26 | **0.039** |
| TR grade pre-interventional, median [IQR] | 3 [1] | 4 [1] | 0.41 |
| TR grade, post-interventional, median [IQR] | 1 [1] | 1 [1] | 0.78 |
| Residual TR grade ≤2, n (%) | 33 (77) | 39 (87) | 0.17 |
| Delta TR grade, median [IQR] | 2 [2] | 2 [2] | 0.56 |
| TR VC, pre-interventional, mm, mean ± SD | 13±4 | 13±4 | 0.78 |
| TR VC, post-interventional, mm, mean ± SD | 4±3 | 4±3 | 0.71 |
| Delta VC, mean ± SD | 9±5 | 9±5 | 0.71 |
| TR Vmax, m/s, mean ± SD | 3.0±0.5 | 3.1±0.6 | 0.39 |
| TR EROA, mm², mean ± SD | 62±33 | 61±21 | 0.90 |
| TR RegVol, ml, mean ± SD | 57±26 | 59±20 | 0.66 |

**TR,** tricuspid regurgitation; **RV,** right ventricle; **TV**, tricuspid valve; **TAPSE,** tricuspid annulus plane systolic excursion; **TDI,** tissue Doppler imaging; **FAC,** fractional area change; **RA**, right atrium; **sPAP,** systolic pulmonary artery pressure; **LVEDV,** left ventricle end-systolic volume; **LV EF,** left ventricular ejection fraction; **LA,** left atrium; **VC,** vena contracta width; **EROA,** effective regurgitant orifice area; **RegVol,** regurgitant volume; **Bold** values are significant.

| Supplementary Table 3 – Medication | | | |
| --- | --- | --- | --- |
| Parameter | **Primary TR** | **Secondary TR** | **P value** |
| Loop diuretic, n (%) | 38 (86) | 271 (91) | 0.23 |
| Aldosterone antagonist, n (%) | 30 (68) | 173 (59) | 0.23 |
| ACEi/ARB, n (%) | 28 (64) | 160 (54) | 0.26 |
| Beta-blocker, n (%) | 34 (77) | 239 (81) | 0.48 |
| Sacubitril, n (%) | 4 (9) | 41 (14) | 0.68 |
| SGLTi, n (%) | 8 (18) | 54 (18) | >0.99 |
| DOAK, n (%) | 33 (75) | 198 (67) | 0.31 |
| Vitamin K antagonist, n (%) | 4 (9) | 60 (20) | 0.10 |

**ACEi**, angio-converting enzyme inhibitor; **ARB**, angiotensin receptor blocker; **SGLTi**, sodium-glucose linked transporter 2 inhibitor; **DOAK** direct oral anticoagulant.

| Supplementary Table 4 – Subgroup analysis of right heart chamber size patterns, right ventricular function, and pulmonary hypertension | | | |
| --- | --- | --- | --- |
| Parameter | **Primary TR** | **Secondary TR** | **P value** |
| RA large (>median), n (%) | 12 (28) | 153 (53) | **0.002** |
| RV large (basal diameter >41mm), n (%) | 26 (59) | 234 (81) | **0.002** |
| RV normal, RA normal, n (%) | 17 (40) | 39 (14) | **<0.001** |
| RV normal, RA large, n (%) | 1 (2) | 16 (6) | 0.49 |
| RV large, RA normal, n (%) | 14 (33) | 95 (33) | >0.99 |
| RV large, RA large, n (%) | 11 (25) | 137 (48) | **0.008** |
| TAPSE <17mm, n (%) | 10 (23) | 130 (49) | **0.002** |
| TR peak velocity >2.8m/s, n (%) | 23 (54) | 162 (57) | 0.74 |

**TR,** tricuspid regurgitation; **RA**, right atrium; **RV,** right ventricle; **TAPSE,** tricuspid annulus plane systolic excursion; **Bold** values are significant.
